# Dopamine-Coated Carbon Nanodots: A Supramolecular Approach to Polydopamine Composite

**DOI:** 10.3390/ijms242015384

**Published:** 2023-10-20

**Authors:** Angelo Nicosia, Placido Mineo, Norberto Micali, Valentina Villari

**Affiliations:** 1 Department of Chemical Sciences, University of Catania, Viale Andrea Doria 6, I-95125 Catania, Italy; angelo.nicosia@unict.it (A.N.); placido.mineo@unict.it (P.M.); 2 CNR-IPCF Istituto per i Processi Chimico-Fisici, Viale F. Stagno d’Alcontres 37, I-98158 Messina, Italy; micali@ipcf.cnr.it

**Keywords:** carbon nanodots, fluorescence, dopamine, supramolecular complex, nanocomposite

## Abstract

The development of biocompatible composites constituted by polydopamine and fluorescent carbon dots represents a promising way of exploiting the extraordinary adhesive properties of polydopamine for multi-purpose technologies. Here, a supramolecular complex is realized by the assembly of dopamine on the carbon dots surface, and the optical and structural properties are investigated by means of different spectroscopic techniques, from time-resolved fluorescence to Raman and NMR spectroscopies. The results suggest that the catechol unit of dopamine plays the main role in the formation of the supramolecular complex, in which carbon nanodot fluorescence emission is quenched by a photoinduced electron transfer process. The interaction with the nanodots’ basic surface sites promotes the oxidation of dopamine and drives to its oligomerization/polymerization on the nanodot surface.

## 1. Introduction

The first steps of the pathway towards polydopamine (PDA) formation are controlled by the pH and are rather well established, starting from dopamine (DA) oxidation and passing through cyclization reactions; although, the structural organization obtained at the end of the process is a topic still under discussion [1]. In particular, it seems accepted that PDA is characterized by heterogeneous structures resulting from covalent polymerization and self-assembly among oligomers and that the oligomers themselves are formed both by uncyclized (dopamine-quinone) and cyclized (dihydroxyindole and dopaminochrome) forms of DA [2,3].

The unique adhesive properties of PDA are stimulating for accessing new materials by means of coating procedures of substrates or particle surfaces, but the interactions underlying the film formation and the contribution of the catechol unit to adhesion are still a debated question, also considering that many PDA films depend on the synthesis conditions [4]. In fact, the large number of active sites offers different interaction mechanisms like hydrogen bonding, electrostatic interactions, π–π stacking interactions, coordination, or chelation [5].

The potential applications of surface modification through wet adhesion technologies with PDA are countless, from bio-interfaces and filtration membranes to self-assembly of nanostructures and composites for electronics, biosensing, drug delivery, nanomedicine, and environmental remediation [6,7,8,9,10,11,12,13].

Combinations of biocompatible components like PDA and carbon dots (or other carbon-based nanostructures [14,15]) offer valuable hints for developing multifunctional composites, exploiting their different characteristics. Recently, for example, many studies have been focused on the coating of carbon nanodots with DA or already-formed PDA to demonstrate the nanodots’ sensing ability towards dopamine and their potential in clinical diagnosis [15,16,17,18,19,20,21]. Moreover, DA-modified carbon nanotubes or fibers succeeded in reinforcing ceramic or polymer composites [22,23,24,25,26].

However, for a better control and modulation of the properties of the obtained composite material, it is important to disclose the interaction mechanisms of DA with the surfaces. In the present work, fluorescent carbon dots are exploited to study the supramolecular interactions of DA with the carbon dots’ surface and the effects on its aggregation/polymerization, since the beginning of the reaction pathway of DA.

## 2. Results

### 2.1. Carbon Nanodots Water Dispersion

**Figure 1:**
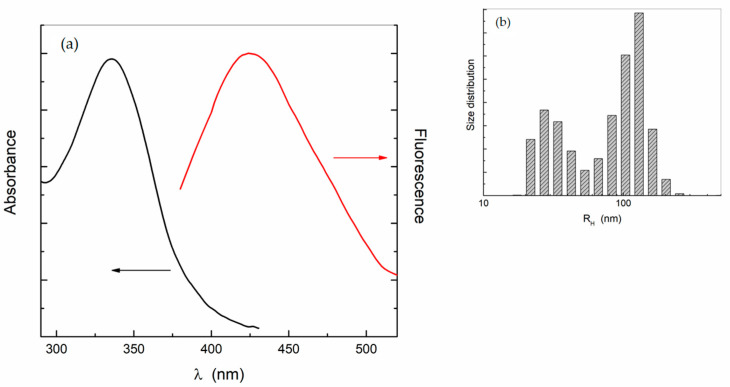
(**a**) Absorption and fluorescence spectra (λ_ex_ = 350 nm) and (**b**) size distribution of the C-dots aqueous solution (0.2 mg/mL).

The nanodots’ water dispersions are characterized by a broad absorption band centered at 335 nm and a fluorescence emission whose wavelength depends on the excitation; a strong emission band appears at 440 nm when excited at λ_ex_ = 350 nm (Figure 1a).

The C-dots’ particle size ranges from 0.5 to 5 nm, as indicated by the seller, and the zeta potential was measured as about −20 mV, by means of Electrophoretic Light Scattering.

The C-dots’ dispersion, however, presents size heterogeneity, as indicated by Light Scattering. It reveals the presence of two families of particles, the main one with an average hydrodynamic radius of about 100 nm and the other of 25 nm, both not evolving with time (Figure 1b). By considering the smaller size of C-dots, these two families point to the presence of clusters, likely due to a low negative surface charge that does not guarantee the necessary electrostatic repulsion between the nanodots.

### 2.2. Dopamine Aqueous Solutions

**Figure 2:**
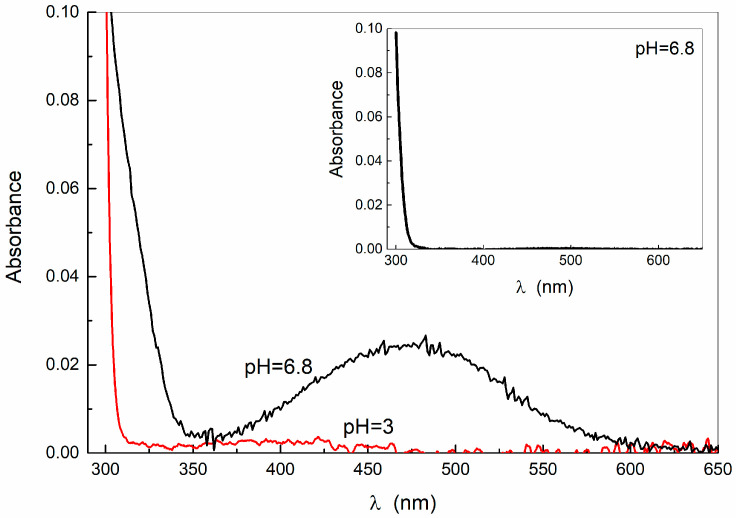
Absorption spectra of DA (0.5 mM) at acidic and neutral conditions. In the inset, the absorbance of DA at pH = 6.8 prepared under an argon atmosphere is reported.

It is well established that DA degradation is triggered by autoxidation due to the dissolved oxygen and accelerated by increasing the pH. The comparison between the absorption profiles of DA aqueous solution at acidic and almost neutral pH, shown in Figure 2, reveals indeed that the band at 290 nm of DA is accompanied by a small band at about 395 nm at pH 3 and a well-defined band at about 475 nm at pH 6.8. The former case can be related to the first oxidation step with the formation of dopamino-quinone [27,28] and the latter to the cyclization reactions and further oxidation steps driving to dopaminechrome [27,29] (see Scheme S1). Such a result is qualitatively in line with a recent thermodynamic model [30], according to which at an acidic pH, the probability of dopaminechrome formation is extremely lower than that of dopamine-quinone, if the amount of dissolved oxygen is the same, likely because the amino group is protected by protonation.

At pH = 6.8 and upon aging for days, the absorbance of the DA solution increases at all wavelengths (Figure S1), with its color turning brownish. On the other hand, at pH = 6.8 in the absence of dissolved oxygen (in argon atmosphere), no absorption band appears above 300 nm (inset of Figure 2), indicating that no mechanism other than oxidation is triggering the reaction.

**Figure 3:**
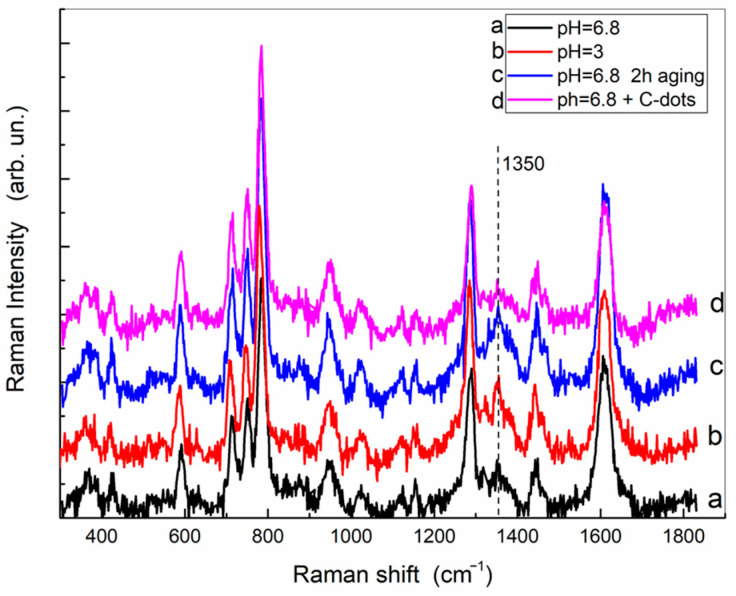
Resonance Raman spectra (vertically shifted for sake of clarity) of DA aqueous solutions (35 mg/mL) collected with excitation at 473 nm. The fluorescence emission background was subtracted. Raw spectra are reported in Figure S2.

Although the Raman spectra do not help to identify the reaction products more precisely, some comments can be made. Figure 3 reports the Raman spectra at acidic and neutral pH, and upon aging. Although the excitation wavelength was selected to be resonant with the dopaminechrome, only the characteristic peaks of DA are present. For instance, the peaks at 1615, 1440, 1285, 950, and 785 cm^−1^ are typically assigned to ring planar and non-planar C–C and C–H deformations as well as C–C stretching of DA [31,32]. No evidence of the C=O stretching mode at about 1680 cm^−1^ [33] is found, which would indicate the presence of dopamine-quinone or dopaminechrome.

Even upon aging, the peak positions remain unaffected, but at pH = 6.8, the background due to fluorescence starts to increase gradually after a couple of hours. The observed fluorescence, despite still being of debated origin, was recently attributed [27] to water-soluble indole-quinone-rich oligomers (see Scheme S2).

In this work, the absence of extended polymerization/aggregation of DA within a day is confirmed via Light Scattering measurements.

Therefore, it appears that the amount of dopamine-quinone, dopaminochrome, or related products remains so low at the beginning of the reaction (and within a couple of hours) that it can be revealed using the absorption measurements and not via Raman scattering. 

It is worth noting that the peak at 1350 cm^−1^, generally appearing with very low intensity in the Raman spectra of dopamine, is quite well defined. The position of such a peak could be assigned to the asymmetric in-plane C-O-H bending (as suggested in ref. [34]),to the C-O stretching of the semiquinone anion [29], or more recently to the CH_2_ wagging of the ethylamine moiety of dopamine [31]. 

The comparison between the spectra of DA at neutral and acidic pH (curves a and b in Figure 3), where no fluorescence emission affects the Raman intensity, indicates that the vibrational mode at 1350 cm^−1^ is better defined at pH = 3 than at pH = 6.8. Under short-time aging at the latter pH value, the relative height of this peak with respect to the closest ones remains the same.

### 2.3. Dopamine–Nanodots Complexes

In the presence of carbon dots, the fluorescence background originating from their emission increases and the background subtraction procedure prevents any evaluation of the absolute intensity, but the inspection of the relative amplitudes indicates that the vibrational peak at 1350 cm^−1^ decreases significantly. This could be interpreted as a clue to the supramolecular interactions of dopamine with the nanodot surface, likely via H-bonding of the catechol group. 

Insights of such interactions can be obtained by inspecting the changes of the structural and optical properties of the nanodots. Indeed, whereas the size distribution of nanodots does not change under the addition of DA in the concentration range investigated, the absorption spectrum displays the appearance of a broad band at 450 nm. The same result is also obtained if the mixing is performed in the absence of dissolved oxygen, under argon atmosphere (Figure S3).

**Figure 4:**
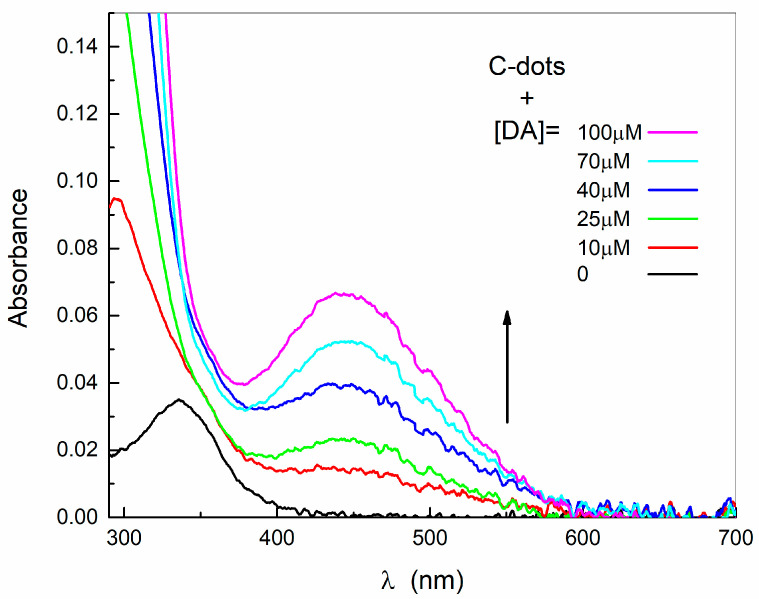
Absorption spectra of nanodots (at 0.2 mg/mL) in the absence and in the presence of DA at different concentrations.

As displayed in Figure 4, this band increases with an increasing concentration of DA because the C-dots act as a reservoir of sites available for interactions and oxidation. Indeed, the band at 450 nm resembles the one observed in a DA solution in the presence of oxygen at a much higher concentration, with a blue-shift of 25 nm, i.e., a concentration of 25 μM of DA in a nanodot solution is sufficient to obtain analogous absorbance at 450 nm of DA alone at 500 μM. The comparison between the absorption spectrum of the same amount of DA in the absence and in the presence of C-dots, reported as an example in Figure S4, indicates that the C-dot surface sites can play the analogous role of the oxygen in the reaction pathway of DA, with a higher efficacy.

Therefore, the nanodot surface basic sites act in place (under argon atmosphere) or along with the dissolved oxygen (in air), favoring the oxidation of DA and triggering (or accelerating) the reaction steps.

**Figure 5:**
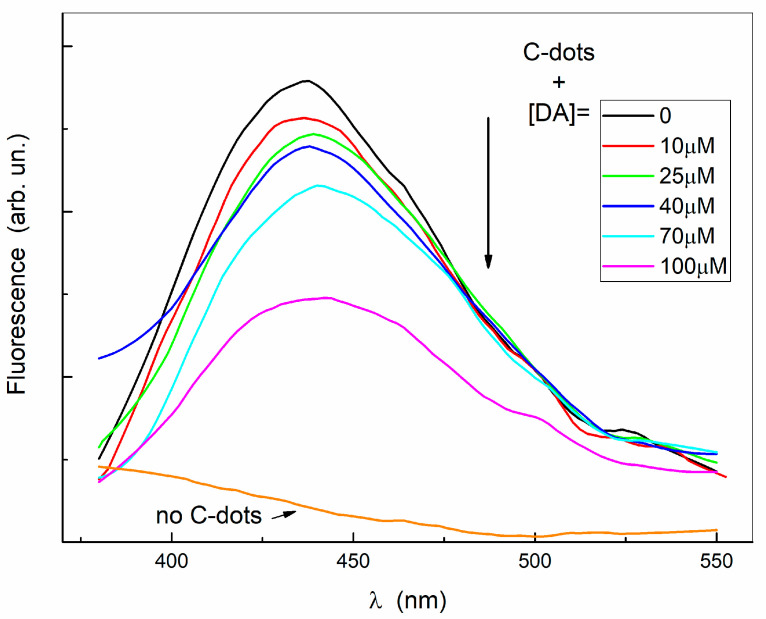
Fluorescence spectra of nanodots (at 0.2 mg/mL) in the absence and in the presence of DA in different amounts (excitation at 350 nm). The spectrum of DA alone is also reported ([DA] = 0.5 mM).

The blue-shift of the absorption band observed above suggests that the cyclized reaction products of DA are experiencing a different environment in the presence of C-dots, reasonably due to the interaction with their surface. To ascertain unequivocally that such an interaction originates from the formation of a stable complex, steady-state and time-resolved fluorescence measurements are performed. Indeed, the fluorescence emission of nanodots at 440 nm is quenched under the addition and increase in the DA amount. The fluorescence spectra of Figure 5 are corrected for the absorbance at the excitation and emission wavelength, excluding the trivial effects of the incident beam attenuation and the inner filter.

**Figure 6:**
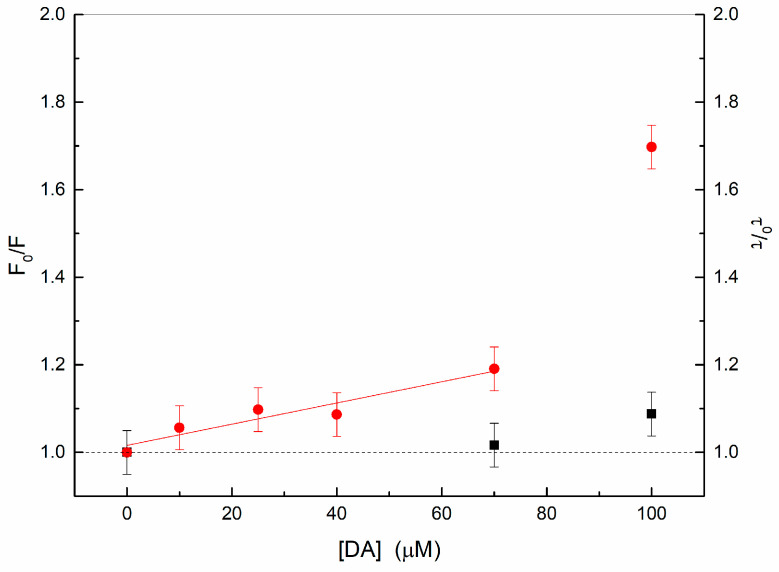
Stern–Volmer plot of the C-dots fluorescence intensity (circles) and the longer lifetime (squares). The continuous line is the fit according to the Stern–Volmer equation.

Below 70 μM, the observed C-dots fluorescence quenching obeys the Stern–Volmer relation (see Figure 6), with a quenching constant of about 2500 M^−1^. On the other hand, the time-resolved fluorescence of C-dots is characterized by three lifetimes of 0.5 ns, 1.5 ns, and 6 ns; these lifetimes, and their relative intensities, remain constant as a function of DA concentration (Figure 6). In the concentration range investigated, DA does not display significant fluorescence emission in comparison with that of the C-dots (Figure 5).

These results suggest the formation of a static adduct between DA and nanodots, with the residual emission being due to the nanodot surface sites that are not yet involved in the interaction with DA.

The bending upwards above 70 μM in the Stern–Volmer plot of the intensity corresponds to a quenching also of the fluorescence lifetimes. In particular, the shorter lifetime blends into the instrumental resolution, whereas the others decrease to 1 and 5.7 ns, respectively. 

Because the fluorescence emission of the DA solutions at 440 nm is negligible with respect to that of C-dots, the decrease in the C-dots’ lifetimes above the DA concentration of 70 μM indicates that another quenching mechanism is occurring independently for the nanodot surface sites not directly involved in the static adduct.

After a couple of hours, precipitation of black insoluble aggregates occurs in the presence of C-dots, at all the DA concentrations investigated. 

Therefore, nanodots do not play a mere catalytic role in the DA oxidation reaction but take part in the aggregation process which starts on the C-dot surfaces and drives towards the formation of a PDA/C-dot composite.

It has to be noticed that because the zeta potential of C-dots does not change significantly in the presence of DA, even at the highest DA concentration investigated, the electrostatic contribution of the ethylamine group at the nanodot surface does not appear to be the main mechanism of DA adsorption [32].

To shed some more light on the aging-related structural modification of DA in the presence of C-dots, NMR experiments were carried out. The supernatant separated from the precipitated after 48 h of aging, both in the absence and in the presence of C-dots, was dried under nitrogen flux and re-dispersed in D_2_O. The resulting ^1^H-NMR spectra compared with that of the fresh DA solution indicate that, in the presence of C-dots, the supernatant is composed only of residual non-interacting DA molecules, or, at least, of non-interacting DA in the very early reaction step (see Figure S5). Therefore, in the presence of C-dots, almost all DA oxidized species evolved towards the further polymerization/aggregation steps and precipitated.

## 3. Discussion

The results from steady-state and time-resolved fluorescence measurements clearly indicate the existence of interactions between DA and nanodots with the formation of stable complexes and the consequent quenching of the C-dots’ emitting surface sites. 

The quenching mechanism of the nanodots can be ascribed to the photo-induced electron transfer in the nanodot/DA complex acting as donor/acceptor system, as observed for other carbon dots [35,36].

The strong decrease in the DA Raman peak at 1350 cm^−1^ suggests that the complex is based on the interaction between the deprotonated catechol ring and the nanodot surface sites [32], reasonably through the C–OH group and/or C–O^-^ anions, and also considering that the invariance of the zeta-potential indicates a not relevant electrostatic contribution of the amino group. 

Hence, the nanodot basic sites accelerate the oxidation steps driving to the oligomerization/polymerization of DA at the nanodot surface. This is consistent with the upward curvature of the Stern–Volmer plot at a higher DA concentration that indicates the action of another quenching mechanism, different from the non-emitting static adduct. Because the diffusive (collisional) model of the dynamic quenching would require a much higher concentration of the quencher in order for it to be adjacent to the fluorophore at the moment of excitation, it is more likely that the nanodot sites not involved in the static adduct experience quenching from more distant interacting DA molecules. In other words, quenching does not occur only at contact, but the quenching rate displays a dependence on distance. Then, it appears reasonable that at a higher DA concentration, some DA molecules can only approach the available nanodot surface sites because of the steric hindrance [35] from other DA molecules already attached or from the presence of DA oligomers triggered by the oxidation due to the interaction with nanodots. 

Although the special wet adhesion properties of PDA on surfaces surely originate from an ensemble of concomitant interactions, the results obtained in this study attribute the main role for the supramolecular complex formation to the DA catechol unit. By considering the high propensity of catechol to interact via hydrogen bonding with different substrates also bearing polar groups [37,38] and its preference to stand perpendicularly to the surface [39], the Raman spectra are consistent with the presence of hydrogen bond-based interactions with C-dots. Due to the almost neutral pH and to the very low amount of the quinone and dopaminechrome forms of DA in solution, such an interaction can lead back to the semiquinone form of dopamine, the very first step of oxidation. The successive oxidation steps of DA and the consequent aggregation/polymerization are, then, triggered at the nanodot surface. 

In addition, the occurrence of the supramolecular complex acting as a donor/acceptor system implies the superposition of the electronic clouds through the π-π interactions. This can occur, considering that the hydroxyl groups of DA can undergo torsions and rotations with respect to the ring to find the right geometry and adapt to the surface [40].

The electrostatic attraction between the protonated amino groups and the negative charges of nanodots, instead, does not appear so relevant at this stage, which could be the rationale for the ability of PDA-based compounds in coating almost all materials. The role of amine groups is indeed acknowledged as crucial in the further steps, for the efficient adhesion and cohesion of the DA/PDA layers during deposition and growth [41,42].

## 4. Materials and Methods

### 4.1. Materials

Dopamine hydrochloride as powder and carbon quantum dots as solution in water (at a concentration of 0.2% *w*/*v*) were purchased from Merck KGaA (Darmstadt, Germany). According to the technical information received by the seller, carbon dots have basic surface sites, have organic-based functional groups (composed of C, H, N, O), and their characteristic size is in the range 0.5–5 nm with D50 = 1 nm. Carbon dots (C-dots) fluorescence emission under UV excitation does not change significantly within 2–3 months from the opening of the bottle; however, for each set of measurements, the fluorescence emission of the nanodots was always collected as reference. 

### 4.2. Sample Preparation 

Two stock solutions of DA at 5 mM were prepared in water, one in air and the other in argon atmosphere, and calculated aliquots were added to the C-dots solution (also prepared under the same conditions as DA) in order for the nanodots concentration to remain constant at 0.2 mg/mL, and DA concentration ranges from 10 to 100 μM. The pH of the prepared solutions is 6.8. Especially for the DA/nanodots solutions prepared in air, care was taken to always use a fresh stock of DA solution. The solution at pH = 3 was prepared by adding a proper amount of HCl.

The NMR spectra of DA were acquired on 100 μM DA solution, using deuterium oxide (D_2_O) as solvent. But, in order to add some information on the effect of DA aging, the solutions of DA in H_2_O (0.1 mg/mL) were prepared, in the absence and in the presence of C-dots (0.2 mg/mL) and aged for two days under stirring in dark conditions at room temperature. The supernatant was then separated from the precipitate and dried under nitrogen flux. The obtained powder residue was re-dispersed in D_2_O (800 μL) and analyzed via ^1^H-NMR spectroscopy. 

### 4.3. Instrument Set-Up

Particle size was obtained by means of the Photon Correlation Spectroscopy technique. The home-made set-up consists of a He-Ne laser source (CVI Melles Griot, Carlsbad, CA, USA), at a power of 35 mW, linearly polarized orthogonal to the scattering plane and focused on the sample, an avalanche photodiode (SPCM, Excelitas Technology, Vaudreuil, QC, Canada) operating in single-photon counting as detector, and a correlator (Malvern 4700, Malvern Panalytical Ltd., Malvern, UK) for the analysis of the scattered light. The CONTIN algorithm was used to obtain the size distribution. More details were reported elsewhere [43].

The particle zeta-potential, ζ, was obtained by the electrophoretic mobility measured with the Zeta PALS instrument (Brookhaven Instrument Corporation, NY, USA), based on the principle of Phase Analysis Light Scattering, through the Henry equation, $\zeta =3\mu \eta /\left[2\varepsilon f\left(\kappa R\right)\right]$. In this equation, *ε* and *η* are the dielectric constant and the viscosity of the solvent, respectively, *κ* is the Debye–Hückel constant, *R* is the particle radius, and 1 ≤ *f(κR)* ≤ 1.5 [44].

The absorption spectra were measured with the fiber-optic Avaspec spectrometer (0.8 nm of resolution) and the fiber-optic Avalight deuterium and halogen source (Avantes B.V., Apeldoorn, The Nederlands).

The Raman spectra were collected by using a home-made equipment consisting of a diode pumped solid state laser at 473 nm (DPSSL, Lasos Lasertechnik GmbH, Jena, Germany) with a maximum power of 50 mW, linearly polarized orthogonal to the scattering plane, a computer-controlled BM100 monochromator (focal 1 m and grating 1200 lines/mm), and a cooled CCD camera (Newton, Andor Technology, Oxford Instruments, Belfast, UK) set at −60 °C. The laser beam passed through a laser line filter and was focused on the sample, and then, the light scattered at 90°, passing through a notch filter to avoid the elastic component reaching the CCD.

Fluorescence emission spectra were collected by the home-made set-up which exploits a Xenon lamp (150 W, Oriel Instruments, Newport, CA, USA), two computer-controlled monochromators, with focal 1/8 m and grating 1200 lines/mm (Oriel Instruments, Newport, CA, USA), for excitation and emission, and an analogic photomultiplier as detector (Hamamatsu Photonics, Hamamtsu City, Japan). The spectral resolution is 4 nm. 

Time-resolved fluorescence was measured by exciting the samples with a polarized femtosecond Ti:Sa laser (Spectra Physics, Newport, CA, USA), with duplication crystal, at 350 nm, without focusing the beam in order to minimize photobleaching effects. The emitted fluorescence was instead focused on the slit of the monocromator (focal 130 mm, holografic grating 1800 lines/mm, Oriel Instruments, Newport, CA, USA) and collected by a microchannel-plate photomultiplier (100 ps rising-time, Hamamatsu Photonics, Japan) operating in single-photon counting mode. Pre-amplification, constant fraction discrimination, Time-to-Amplitude Conversion (TAC), and acquisition of time-resolved fluorescence curves were obtained by EG&G electronic devices (Ortec EG&G, Ametek Inc., Berwyn, PA, USA). The fitting of the total fluorescence curves was performed through a nonlinear least-squares iterative reconvolution, according to a multiexponential law, $I\left(t\right)=I_{0}\sum_{i}A_{i}exp\left(t/\tau_{i}\right)$, with *I*_0_ being the fluorescence intensity at time zero, and *A_i_* and *τ_i_* being the relative intensity and lifetime of the *i*-th component, respectively, and $\sum_{i}A_{i}=1$. With such a procedure, the obtained instrumental resolution is few tens of picoseconds. More details were reported elsewhere [45].

^1^H-NMR spectra were acquired using a ^UNITY^INOVA (Varian, Agilent Technologies, Santa Clara, CA, USA) operating at 500 MHz (^1^H). Spectra acquisition and processing were performed at 27 °C using VnmrJ software (Version 6.1C). The chemical shifts were expressed in ppm.

### 4.4. Static and Dynamic Fluorescence Quenching

In the absence of resonance energy transfer, which implies a through-space interaction, the fluorescence quenching requires molecular contact between the fluorophore and quencher. This quenching can be dynamic for diffusive encounters or static in the case of the formation of complexes. According to the Stern–Volmer approach, it is

(1) $$\frac{F_{0}}{F}=1+K_{SV}[Q]$$

(2) $$\frac{\tau_{0}}{\tau }=1+K_{D}[Q]$$

in which *F*_0_ and *τ*_0_ are the fluorescence intensity and lifetime, respectively, of the fluorophore alone, *F* and *τ* are those in the presence of a different concentration of quencher, [*Q*], *K_SV_* is the Stern–Volmer quenching constant, and *K_D_* is the dynamic one.

For a pure static quenching, Equation (1) holds, with *τ*_0_/*τ* = 1; in other words, the complex formed does not emit the residual fluorescence due to the free fluorophores. For a pure dynamic quenching, instead, the condition $\frac{F_{0}}{F}=\frac{\tau_{0}}{\tau }=1+K_{D}[Q]$ is observed.

In many real cases, however, the quenching is not purely static or purely dynamic. Sometimes an upward curvature of the fluorescence intensity quenching could indicate a mixed static/dynamic quenching or a distance-dependent quenching, the latter being characterized by a rate of quenching exponentially dependent on the fluorophore-quencher distance [46].

## 5. Conclusions

PDA coating on substrates or nanoparticle surfaces has been stimulating many studies thanks to the very peculiar adhesive properties of PDA on almost all materials. Carbon nanodots represent a good challenge for studying fluorescent biocompatible composites, exploiting the PDA properties. Although DA molecules can display diversified interaction mechanisms, by using different spectroscopic techniques, it was shown that the formation of the supramolecular complex between the carbon nanodot and DA occurs mainly via hydrogen bonding, through the catechol unit. The basic surface sites of carbon nanodots cause the oxidation of DA and the beginning of the reaction pathway towards the PDA formation, with the consequent coating of the nanodot surface. 

In the special wet adhesion properties of PDA, many other factors must be surely taken into account which have not been considered here, but the leading idea of this work is to disclose information on the interaction mechanisms characterizing the very early steps of the PDA reaction pathway. This approach can allow for improving the ability of modulating the characteristic and properties of the composites for technological and biocompatible materials and also for applications in new generation photovoltaic devices.

## Data Availability

The data presented in this study are available in this article and in the Supplementary Material.

## Supplementary Materials

The following supporting information can be downloaded at: https://www.mdpi.com/article/10.3390/ijms242015384/s1.
