# Supplementary material for: Dopamine-Coated Carbon Nanodots: A Supramolecular Approach to Polydopamine Composite
Source: Int J Mol Sci. 2023 Oct 20;24(20):15384. doi: 10.3390/ijms242015384 (PMC10607924; doi:10.3390/ijms242015384)
Supplement: Supplementary file 1 [file ijms-24-15384-s001.zip › ijms-2648887-supplementary.pdf]

Supplementary Material

Dopamine-coated Carbon Nanodots: a supramolecular approach to Polydopamine composite

Angelo Nicosia [1], Placido Mineo [1,2], Norberto Micali [2] and Valentina Villari [2,*]

[1] Dipartimento di Scienze Chimiche, Università di Catania, Viale Andrea Doria 6, I-95125 Catania, Italy
[2] CNR-IPCF Istituto per i Processi Chimico-Fisici, Viale F. Stagno d'Alcontres 37, I-98158 Messina, Italy
* Correspondence: villari@ipcf.cnr.it

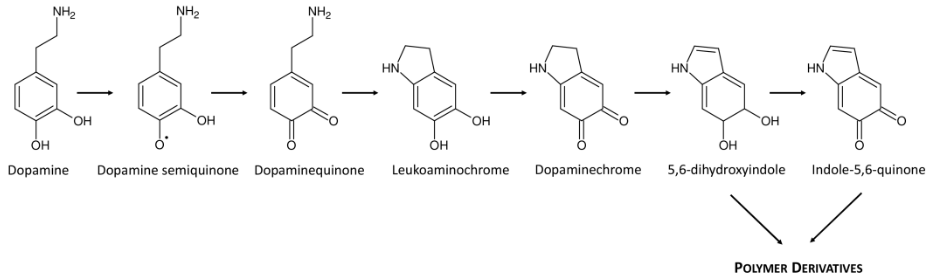

Scheme S1. Oxidation pathway of Dopamine.

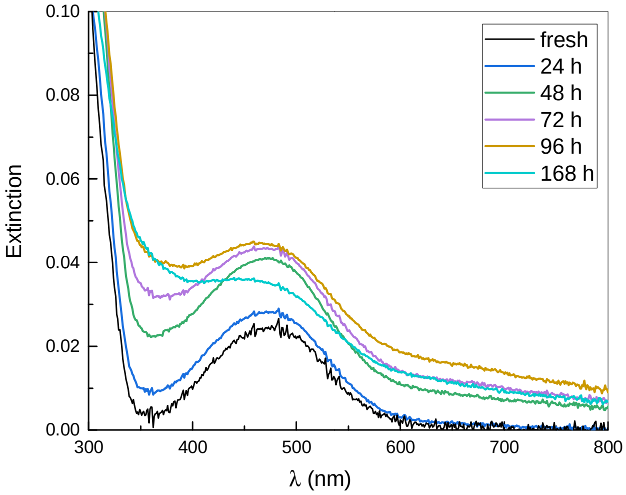

Figure S1. Extinction spectra of DA solutions at pH=6.8 upon days of aging ([DA]=0.5 mM). Within 24 hours from preparation no significant changes are observed in the absorption profile apart from a slight increase of the absorbance at 465 nm, indicating that the oxidation process is ongoing. At 48 hours and over, the beginning of the aggregation/polymerization is

indicated by the increase of the absorbance in all the wavelength range, whereas after a week the precipitation of insoluble PDA is occurring.

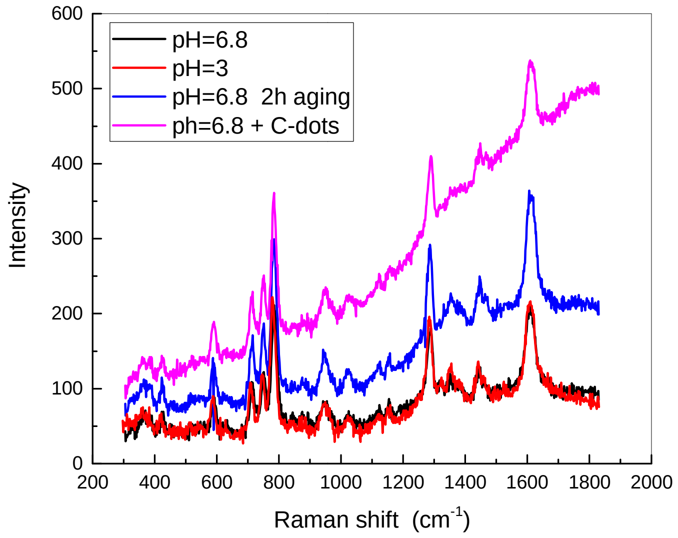

Figure S2. Raman spectra of dopamine under different solution conditions. The background is due to fluorescence emission.

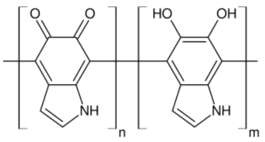

Scheme S2. Proposed structure of fluorescent dopamine-based oligomers (3≤ (n+m)≤5).

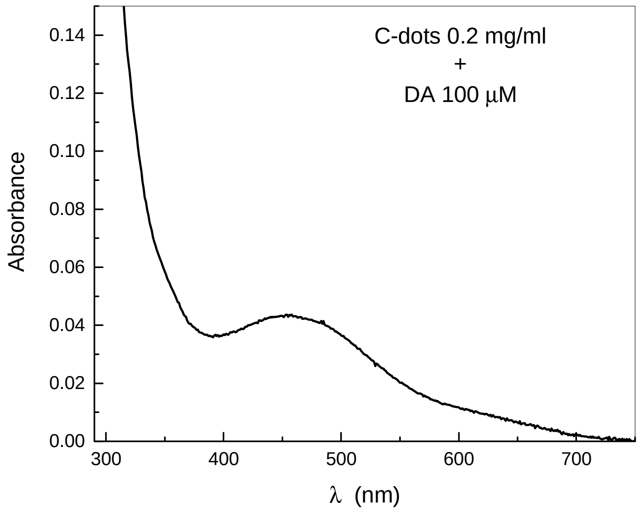

Figure S3. Absorption spectrum of DA/C-dots solution at pH=6.8 prepared under argon atmosphere.

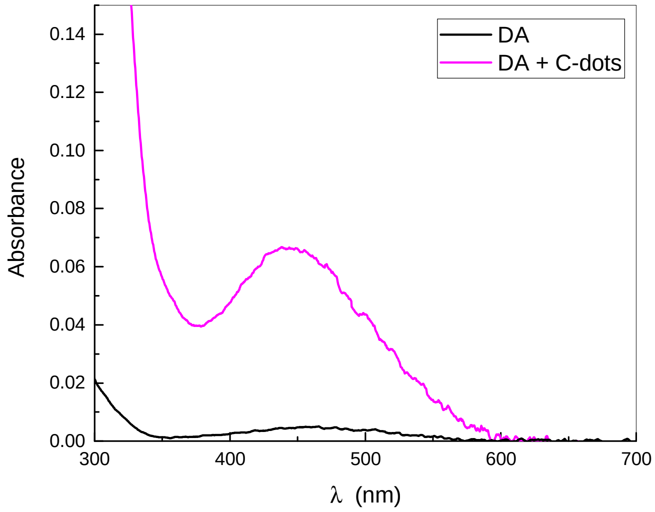

Figure S4. Comparison between the absorption spectrum of DA and DA/C-dots solution at pH=6.8 in air. The concentration of DA is 100 μM and that of C-dots 0.2 mg/ml.

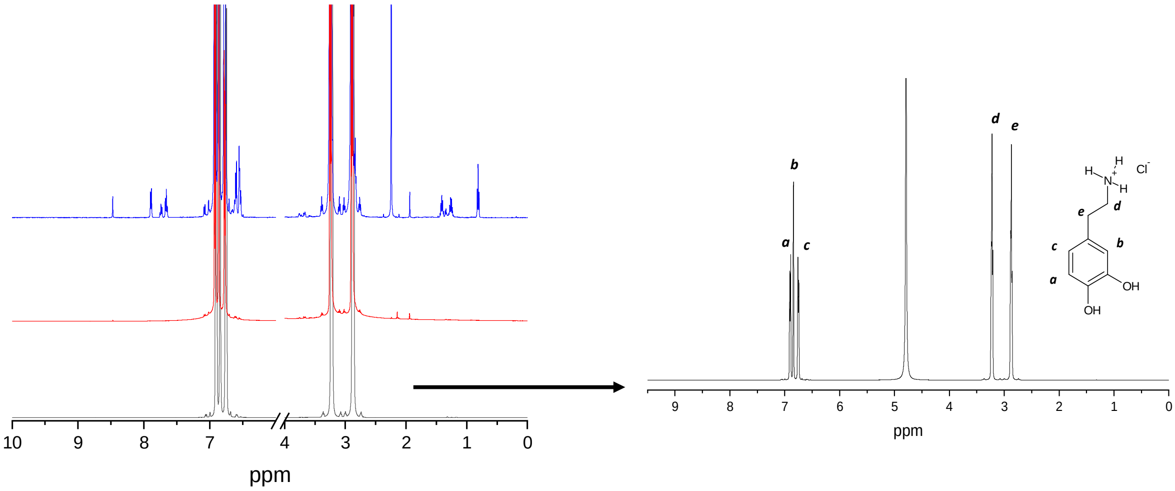

Figure S5. ^{1}H-NMR of fresh DA (black line), supernatant aged DA+C-dots (red line) and supernatant aged DA (blue line) in D_2O. In the inset, the attribution of the peaks of the fresh DA is reported. The ^{1}H-NMR of fresh DA shows the following peaks: *a*, 6.90 ppm, 1H; *b*, 6.84 ppm, 1H;*c*, 6.75 ppm, 1H;*e*, 3.23 ppm, 2H; *d*, 2.88 ppm, 2H. The spectra of the supernatant of aged DA shows many additional peaks with respect to fresh DA. Among these, the signals at 2.84, 6.55, 6.60 ppm could be attributed to the Dopamine-o-quinone, according to DA oxidation. The appearance of the signals at ppm values in the range 8.5 - 7.5 is attributable to the hydrogen atoms at the end-groups and to the indole groups of the polymerized indole-quinone and/or 5,6-Dihydroxyindole–oligomeric species [Bisaglia, M.; Mammi, S.; Bubacco, L., Kinetic and Structural Analysis of the Early Oxidation Products of Dopamine. *J. Biol. Chem.* **2007,** 282, (21), 15597-15605]. Furthermore, the presence of multiplets at about 1.3 ppm suggests the deamination of dopamine (and of its oxidate derivatives). Finally, the singlet at about 2.3ppm suggests the formation of di-hydroxytoluene and/or 4-Methyl-o-benzoquinone species. All these peaks do not appear in the supernatant of aged DA+C-dots. The comparison is only qualitative and cannot tell anything about the amount of DA in the supernatant, but it clearly suggests that, in the presence of C-dots, almost all DA oxidated species evolved towards the further polymerization/aggregation steps and precipitated.
